# The Anthrax Order

**Published:** 1921-07-02

**Authors:** 


### THE ANTHRAX ORDER.

The popular scare and the amusing suggestions as to how best to disinfect yet not destroy our shaving-brushes are now past history. But it is interesting to remember some of the real facts in connection with the scattered cases of anthrax which occurred before the Government took extra precautions against these manufactures from Japan. During 1920 an interesting series of human infections was traced; there were twenty-one different parts of the country, London claiming six of them. There were six deaths, and in every case the site of the malignant pustule was definitely in the shaved area. In seventeen cases the diagnosis of anthrax was fully confirmed bacteriologically, and in fifteen the germ was also found in the suspected brushes.

At first sight, and in a hospital clinic, the sequence of events may seem simple, but the difficulties of tracing the source and likely ramifications of such an outbreak are by no means light. As an instance, we may recall a consignment of 650 gross sold to one wholesale firm in England, and these resold in lots to ten other wholesale firms, which, in their turn, resold in small quantities to over 100 retail firms trading not only in the United Kingdom, but all over Europe and Asia. Some of the brushes were consigned actually wrapped in sheets of paper marked "Sterilised and free from anthrax," and it is said that some of the batches were even accompanied by an official document from the Japanese signed and sealed to the same effect!

There was indeed ample justification for the action taken by the Ministry of Health in issuing an Order in Council under the Anthrax Prevention Act of 1919, prohibiting for the time being further importation into the United Kingdom of shaving-brushes manufactured in, or exported from, the Empire of Japan. This Order came into force in February 1920, and the warning notices in the press were then familiar to us all. Extremely large numbers of brushes were hunted down or surrendered, and indeed the search for anthrax in this direction still continues. The amount of work involved has been far greater than the public may believe. The cases have, it is true, not been really alarming in their number, but if one has ever seen an acute anthrax infection it is remarkably easy to realise full justification for the time and trouble spent on this problem by the Ministry and its officers.

